# On the Use of Cathartics in Retention of the Placenta

**Published:** 1843-05

**Authors:** Thomas H. Todd

**Affiliations:** Lincoln county, Tennessee


March, 1843.

---

Art. V.—*On the Use of Cathartics in Retention of the Placenta.* By Dr. Thomas H. Todd, of Lincoln county, Tennessee.

Although much has been said and written on the subject of retained placenta, yet we have much to learn about it, as indeed about many things in every other branch of medicine. It is not my design to write an elaborate dissertation upon this topic. My aim, in the remarks that I shall make, will be, to direct the minds of practitioners to a certain class of remedies which have heretofore, so far as my information extends, been overlooked, or entirely neglected by the practical accoucheur. I must therefore refer the reader to the standard works upon obstetrics, and to the numerous essays contained in the medical journals, for a full account of the subject, and the general management of such cases; while I shall confine my remarks to the therapeutic agency of *cathartic medicines*, as a means of expelling the secundines under certain circumstances. Why cathartics have so long been overlooked and neglected by the profession, is really matter of much surprise to me; for we cannot peruse the writings of any one upon this subject, without being able to glean abundant evidence, that goes conclusively to establish the efficacy of this class of medicines, in rousing up the dormant energies of the uterus. And they will do this, independent of any particular irritation or excitement which they may produce upon the rectum. The older writers on midwifery discovered that there was an intimate sympathy existing between the rectum and uterus, and that any undue excitement in the former, was readily communicated to the latter organ: hence, they early learned the efficacy of stimulating injections in cases where they desired uterine contraction. To the correctness of this fact all subsequent writers have testified. But why this discovery should forestall all further research and inquiry, respecting the sympathy that might exist between the uterus and the bowels, instead of leading to it, is a problem that I shall leave for others to solve. In the meantime, however, I shall endeavor to prove that this sympathy is not confined to the rectum, but extends to the whole alimentary canal.

All writers in treating of the diseases of the pregnant female are unanimous in cautioning us in the use of cathartics, lest, say they, the use of this class of remedies should excite the uterus, and thereby produce abortion. Now it is well known that *drastic cathartics* when carried to excess, are prone to excite the rectum, and to produce tenesmus; but that all purgative medicines, when not carried too far, will produce this effect, no one will hazard his reputation by asserting. The late Dr. Dewees says: "The pregnant woman does not bear purging so well as one who is not so, and if this operation be carried very far, there is a risk of producing abortion." In speaking further upon this subject he says: "We suggest a caution in the choice of purgative medicines; all such as act with great force upon the bowels should be avoided; as all such as are classed among the drastic purgatives, as scammony, gamboge, colocynth, aloes, &c.; because each of these produces, during its operation, great irritation and very frequently excites tenesmus. It is this *peculiar irritation* which renders any cathartic unsafe which may produce it; no matter to which class of cathartics it may belong; for if castor oil, magnesia, or any other mild cathartic were to produce this effect, it would be equally improper, as any of the drugs proscribed above."

The foregoing quotation serves to show that any purgative will sometimes produce uterine contraction: but Dewees thinks it will only do so, by first producing a *peculiar irritation* upon the rectum. He has not, however, attempted to prove that castor-oil, magnesia, or any other mild cathartic, ever does produce this peculiar irritation upon the rectum, when used in moderation. There is no one who appreciates the opinions of eminent men, and those in high places, more than the writer does; but before we admit them as maxims in science, we ought at least to examine the premises upon which they are founded. That uterine contraction, when there is a predisposition in this organ, frequently follows, or accompanies the action of the bowels produced by any mild purgative, is manifest to all who have been in the habit of observing their effects upon the parturient female. I see nothing connected with the uterus, and its sympathies, but what goes to corroborate this fact. When the uterus is predisposed to contract, as it always is during the retention of the after-birth, we see this action excited by the slightest causes, as gentle traction upon the cord, friction over the abdomen, the application of a cold or wet hand, &c. After observing the effects produced by such slight causes, why should we be so incredulous as not to believe that an action upon the bowels, sufficient to cause them to throw off their contents, could not make an impression upon so excitable an organ as the uterus is at such times, independent of producing any particular irritation, or tenesmus about the rectum. Denman, in speaking of the causes of abortion, says: "The sympathetic causes of the action of the uterus may arise from the disturbance of any part, with which the uterus is connected, or disposed to consent, as is the case with *all the contents* of the abdomen." Again he says; "Pain in the stomach, or bowels, or any part contiguous to the uterus, or with which it is prone to consent, may disturb it; and if extremely violent, or of long continuance, may produce the same effect." And again he continues: "Such medicines as exert a violent action on the stomach or bowels, will, upon the principle formerly mentioned, frequently excite abortion; and very often are taken designedly for that purpose."

In the American Journal of the Medical Sciences (vol. 26, no. for May, 1840), Dr. Edward Warren, of Boston, has written a valuable paper on retention of the placenta. His aim is to prove, by correct data, that there is less danger in delay, in such cases, than in the violent means generally resorted to, for the speedy removal of the secundines. Among the cases cited in this essay, are the two following, taken from a report of Dr. Lee's: "In the seventeenth case, the woman was delivered of a child at the sixth and a half month. The cord had been broken off by the midwife, and the parts were so much contracted that the hand could not be introduced without too much force. The following morning a brisk cathartic was given, and in the evening the placenta came away whole, without any help, and with no bad result. In the nineteenth case, the patient had been delivered of a dead child, of six and a half months, twenty-four hours before Dr. Lee saw her. An attempt to withdraw the placenta failed. The next morning a cathartic was given, which excited vomiting and purging, and during its operation the placenta came away in a yellow indurated state—no bad symptom followed." This case is directly in point. In case seventeenth, a brisk cathartic was given in the morning, and the placenta came away in the evening; by which time, the cathartic must have been operating. I have very little doubt that the cathartic excited the uterus in this case, although the Dr. tells us it came away whole without any help.

During my residence in Starkville, Mississippi, the following cases came under my own observation:

*Case 1st.* A woman aged twenty-eight, the property of

Richard Ellett, was delivered of a living, and *first* child. The labor was tedious, and the patient much exhausted in consequence. After the child was removed, I examined the parietes of the abdomen, and found that the uterus had contracted firmly; but the tumour was high up in the right side. As there was no hemorrhage, I used friction, and pulled gently upon the cord only, for the first few hours. I then passed my fingers along the cord to ascertain if the placenta had been detached and thrown into the vagina; but it had not, as I was unable to touch any part of it. I then administered the ergot, with the view of exciting uterine contraction, and thereby relieving the patient; but in this I was disappointed, as the ergot produced no effect. After this failure I thought it best to introduce the hand and remove the after-birth, but here again I was doomed to meet another disappointment; as the soft parts had by this time contracted so much, that any attempt to introduce the hand, however slow and firm it might be, entirely failed. I now resolved to give a dose of ol. ricini, as the bowels had not been moved since the labor began, and to wait the result. In due time, however, the oil acted, and with the action of the bowels the pains returned, and threw the secundines into the vagina, where the mass was readily seized, and removed with the hand twelve hours after the birth of the child.

*Case 2d.* Prissa, servant of Mr. Hines, was delivered of a still-born child near the sixth or seventh month (in September, 1839). On examination twelve hours after delivery, I found that the cord had been broken off in attempts to remove the after-birth. The parts contracted so much that the hand could not be introduced; and the ergot had been given but produced no effect. As the bowels had not been moved, I advised that a dose of castor-oil should be given, and its operation waited for, as there were no unpleasant symptoms in the case. By evening the oil had operated two or three times, without producing any pain whatever. I now made an examination, but found that the placenta had not advanced any. As the patient was resting well, we resolved to leave the case for the night to nature. At 10 o'clock next morning we gave a dose of pills containing equal parts of calomel, rheubarb and aloes. At 6 o'clock, P. M., I was sent for in great haste; the woman was in much pain and her mistress much alarmed. Soon after my arrival the pills acted upon the bowels; and with the first evacuation the placenta was expelled, forty six hours after the birth of the child.

These two cases were communicated to the editor of the American Journal of the Medical Sciences, and published in vol. ii (new series) no. 3, for July, 1841; with the following paragraph by the editor: "The well known sympathy between the rectum and uterus, explains the action of purgatives in exciting the uterus to expel its contents." This paragraph is a fit illustration of the influence that preconceived opinions and prejudice always exert over the minds of men. Now, if there is any thing, in either of the cases related above, that could lead us to conclude that there was any preternatural excitement, or tenesmus, in the rectum, it is hidden from the writer. In the first, the bowels had not been moved previously by any medicine, and oil was used on the occasion. In the second, the bowels had not been operated upon for the last twenty-four hours, and the placenta was expelled after the first evacuation.

*Case 3d.* I was called, in November, 1840, to see a woman the property of Richard Barry. She had been delivered twenty-four hours before I was called. The placenta had not been delivered, and the cord had been broken off by the midwife. The soft parts were so much contracted, and so tender to the touch, that I could not introduce the hand, without using too much violence. I gave ergot in full and repeated doses; but without producing any effect upon the uterus. As the bowels had not been opened, I gave a dose of Cook's pills. By evening they operated, producing considerable pain in the uterus; but to make sure work, I resumed the use of the ergot at this time; and in a little while the placenta was expelled. No unpleasant symptoms followed.

*Case 4th.* This case I did not see, on account of my own ill-health at the time. The patient, however, was my immediate neighbor, and was treated by my brother, J. J. Todd, M. D., of Starkville, Mississippi. Mrs. F. a delicate female, and the mother of one child, miscarried in July, 1841, between the fifth and sixth months. The after-birth could not be delivered at the time, although ergot was used for that purpose. Next day the cord was broken off, in unsuccessful attempts for its removal. A purgative had been given to open the bowels, and when it acted, ergot was again used, but without producing any effect. As the case was not attended with any unfavorable symptoms, no rash means were resorted to. Early on the third day however, during the action of her bowels from a purgative dose of medicine, the placenta was expelled—without any help, as some writers would say. No bad symptom occurred afterward.

It frequently happens, that all remedies that can be brought to our aid, fail; and the accoucheur has at last to leave the case almost entirely to the efforts of nature. Friction and pressure upon the abdomen fail, ergot fails, traction upon the cord fails, the hand cannot be introduced with safety, injections do not relieve, nor will instruments answer our purpose. And yet suffering humanity calls incessantly upon us for relief. Under these circumstances we ought surely to search out, and call to our aid, each and every remedy that will tend to relieve the patient, without any detriment. Purgatives, to say the least for them, certainly exerted a salutary influence in the cases above mentioned, and that, after all other safe means had been resorted to without success.

